# Evaluation of commercial susceptibility testing methods for *in vitro* determining daptomycin susceptibility in vancomycin-resistant *Enterococcus faecium*

**DOI:** 10.1128/spectrum.03590-25

**Published:** 2026-03-05

**Authors:** Stefano Mancini, NARA Network members, Dominique S. Blanc, Silvio D. Brugger, Sari Rasheed, Markus Jutzi, Natalia Kolesnik-Goldmann, Rolf Mueller, Adrian Egli, Vladimira Hinic, Oliver Nolte

**Affiliations:** 1 Institute of Medical Microbiology, University of Zurich 27217, Zurich, Switzerland; 2 Institute of Medical Microbiology, Lausanne University Hospital and University of Lausanne, Lausanne, Switzerland; 3 Swiss National Reference Center for Emerging Antibiotic Resistance, Fribourg, Switzerland; 4 Department of Infectious Diseases and Hospital Epidemiology, University Hospital Zurich, University of Zurich 27243 , Zurich, Switzerland; 5 Helmholtz Institute for Pharmaceutical Research Saarland (HIPS), Helmholtz Center for Infection Research (HZI), Saarland University 9379, Saarbrücken, Germany; 6 German Centre for Infection Research (DZIF), Partner Site Hannover, Braunschweig, Germany; 7 Analytica Medizinische Laboratorien AG, Zurich, Switzerland; Wannan Medical College, Wuhu, Anhui Province, China

**Keywords:** gradient diffusion test, broth microdilution, daptomycin AST, vancomycin-resistant *Enterococcus faecium*

## Abstract

Vancomycin-resistant *Enterococcus faecium* (VR*Efm*) is a major cause of invasive healthcare-associated infections. Daptomycin is an important treatment option, but its efficacy may be compromised for isolates with minimum inhibitory concentrations (MICs) of 4–8 mg/L, even at high doses. EUCAST has therefore assigned an “insufficient evidence” category for *Enterococcus* spp., making accurate MIC determination essential. We compared the performance of UMIC Daptomycin, several automated broth microdilution (BMD) systems, and E-test against the reference BMD method. Eighty-nine VR*Efm* isolates with borderline daptomycin susceptibility were tested by standard BMD in calcium-adjusted Mueller–Hinton broth and by Phoenix, EUVENC, VITEK-2, UMIC Daptomycin, and E-test. CLSI breakpoints (susceptible-dose dependent ≤ 4 mg/L, *R* > 4 mg/L) were applied. Essential agreement (EA), categorical agreement (CA), bias, very major errors (vMEs), and major errors were assessed. Phoenix (EA = 92%, CA = 69%) and EUVENC (EA = 87.6%, CA = 74.2%) were the most accurate. UMIC achieved EA = 74.2% and CA = 70.8%. E-test (EA = 68.5%, CA = 66.3%) and VITEK-2 (EA = 67.4%, CA = 64%) showed lower accuracy. All methods displayed negative bias, underestimating MICs and frequently misclassifying isolates with MIC = 8 mg/L, resulting in high vME rates. None of the methods met ISO-20776-2:2021 criteria. Automated BMD systems performed best, but all showed systematic MIC underestimation near the breakpoint. For reliable detection and confirmation of daptomycin resistance in VR*Efm*, BMD-based MIC methods—manual or automated—are recommended.

**IMPORTANCE:**

Vancomycin-resistant *Enterococcus faecium* (VR*Efm*) is a major cause of hospital-acquired infections worldwide, posing a serious threat to patient safety and infection control. Daptomycin remains one of the few therapeutic options for severe VR*Efm* infections, yet its efficacy depends on accurately determining bacterial susceptibility. Even small variations in measured minimum inhibitory concentrations can influence treatment success or failure. In this study, we systematically evaluated several commercial antimicrobial susceptibility testing methods for daptomycin against VR*Efm* and compared them with the reference broth microdilution (BMD) method. Our results show that many commonly used systems underestimate resistance, particularly for isolates near the clinical breakpoint. This underestimation may lead to inappropriate treatment decisions. The study provides evidence-based recommendations for clinical laboratories and emphasizes the importance of confirmatory BMD testing to ensure reliable results, optimize patient management, and prevent the further spread of antimicrobial resistance.

## INTRODUCTION

Rising rates of vancomycin-resistant *Enterococcus faecium* (VR*Efm*), for which treatment options are limited, are globally reported (1). In this setting, daptomycin has emerged as an important treatment option for invasive infections caused by VR*Efm,* including bacteremia, endocarditis, complicated skin and soft tissue infections, and osteomyelitis (2). However, uncertainties remain, particularly regarding the inability of even the highest published doses (12 mg/kg/day) to achieve adequate exposure against all wild-type isolates (with minimum inhibitory concentrations [MICs] of 4 or 8 mg/L) (3). Due to these concerns, EUCAST has not proposed clinical breakpoints (CBPs) for daptomycin and *Enterococcus* species but rather listed the breakpoint as “IE” = insufficient evidence (4). In contrast, CLSI defines susceptible-dose dependent isolates as those with MIC ≤ 4 mg/L and resistant isolates as those with MIC ≥ 8 mg/L (5). Moreover, several studies have reported intra- and inter-method variabilities as well as issues with the reliability of laboratory testing, which may impact treatment outcomes, particularly in cases of borderline susceptibilities (6, 7). Finally, increasing MICs have been linked to rising daptomycin use in several reports (8). While broth microdilution (BMD) is the gold standard, its use in clinical laboratories is unsuitable due to the labor-intensive nature of the method and the technical challenges to maintain consistent Ca^++^ concentrations (50 mg/L) for reproducible results (9). Since Ca^++^ concentration is difficult to control in Mueller–Hinton agar, disk diffusion is also unsuitable. Beyond commercial BMD methods, gradient diffusion strips are widely used in routine diagnostics, as according to the manufacturers’ instructions, they provide consistent Ca^++^ concentrations within the strip. However, media and operator-dependent variability have been reported (10). Given the critical role of MIC determination and uncertainty regarding the reliability of antibiotic susceptibility testing (AST), evaluating commonly used diagnostic methods may help identify the most reliable approach. UMIC Daptomycin, a manual BMD method recently developed by Bruker (Massachusetts, USA), is specifically designed for diagnostic laboratories that do not rely on automated systems for AST, such as those using disk diffusion. However, data on its performance with *Enterococcus* species are currently lacking, highlighting the need for further evaluation.

Using a large collection of VR*Efm* isolates with borderline daptomycin susceptibility, we aimed to compare UMIC Daptomycin, several widely used commercial BMD methods, and a gradient MIC strip with the gold standard BMD method and assess their performance.

## MATERIALS AND METHODS

### Strain collection

A collection of 89 VR*Efm* clinical strains, predominantly with borderline daptomycin susceptibility, each isolated from individual patients between 2019 and 2024, was provided from the Swiss National Reference Center for Emerging Antibiotic Resistance. The isolates were specifically selected to be close to the clinical break point to better highlight potential differences between methods. *Enterococcus faecalis* ATCC 29212 was used as the internal quality control strain.

### Susceptibility testing

The reference method was manual BMD in cation-adjusted Mueller–Hinton (CAMH) broth supplemented with 50 mg/L Ca^++^, following both EUCAST and CLSI guidelines (5). The following commercial BMD methods were performed according to the manufacturers’ instructions: the automated BMD AST systems (i) Sensititre using the *Enterococcus* EUVENC Sensititre plate (Thermo Fisher), (ii) Phoenix using the PMIC/ID-88 plate (BD Diagnostics), (iii) VITEK-2 using the P-636 card (bioMérieux), (iv) the manual BMD strip UMIC Daptomycin (Bruker), and (v) E-test (bioMérieux). The E-test was performed on regular CAMH agar plates from bioMérieux (Marcy L’Etoile, France). Testing was conducted across participating laboratories. Bacterial suspensions equivalent to a 0.5 McFarland standard according to EUCAST recommendations (11) were prepared independently at each site, and the QC *E. faecalis* ATCC 29212 was included in every run. Runs were accepted only when QC results were within the CLSI-recommended daptomycin MIC QC range (1–4 mg/L) (5). Raw data are reported in Table S1.

### Data analysis

We used descriptive statistical analysis. We categorized the bacterial isolates based on standard BMD-derived MICs and the CLSI CBPs (SSD ≤ 4 mg/L, *R* > 4 mg/L) (5). We determined the categorical agreement (CA), essential agreement (EA), bias, very major errors (vMEs), and major errors (MEs) for each AST method.

## RESULTS

**Fig 1:**
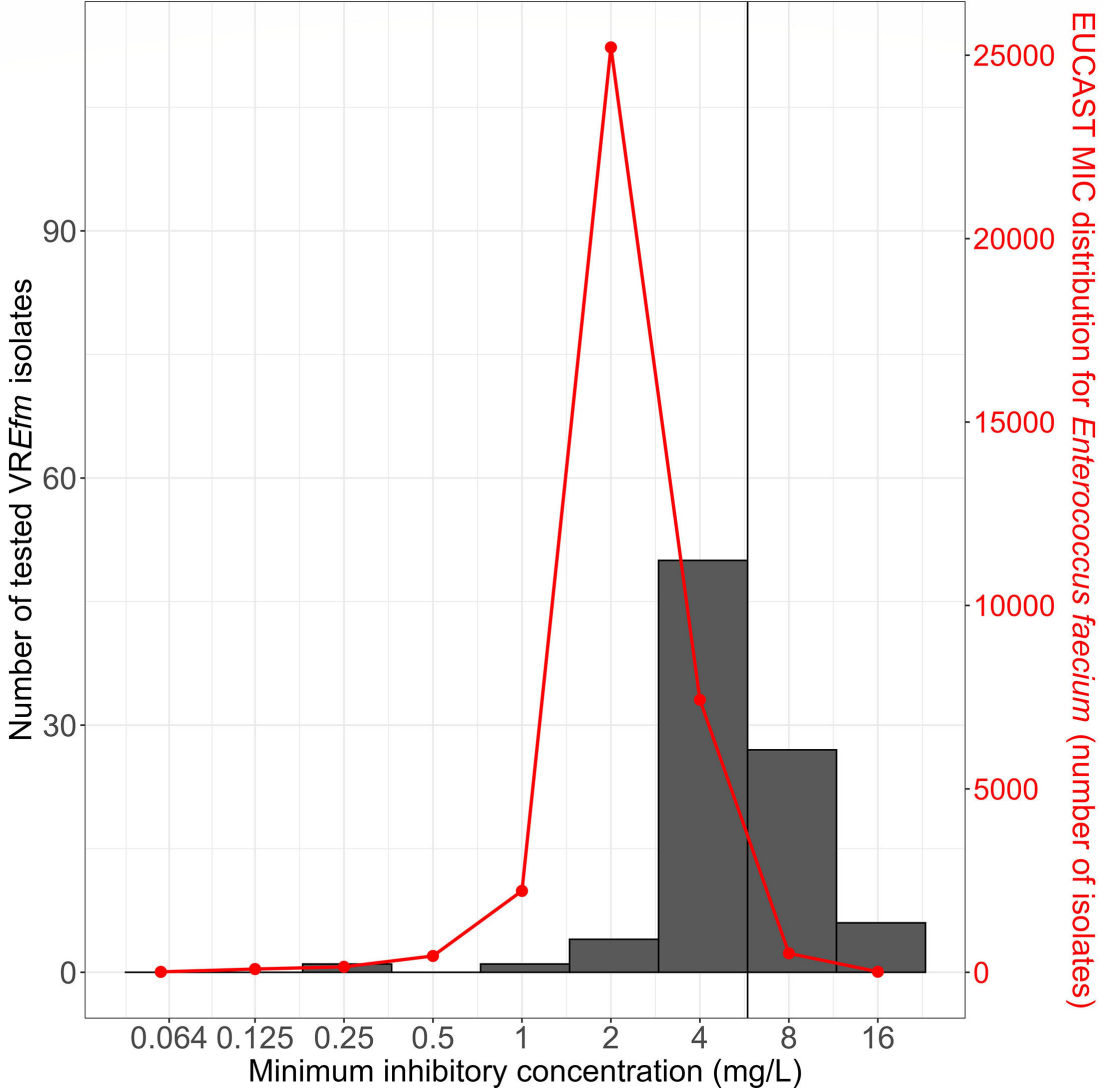
Distribution of daptomycin MICs. The bars indicate MICs determined by the gold standard BMD according to the EUCAST guidelines. The red line reflects the EUCAST MIC distribution for *E. faecium*. The vertical line denotes the CLSI CBP for resistance (>4 mg/L).

Most isolates (80/89, 90%) exhibited gold standard BMD MICs within ±1 fold dilution of the CLSI CBP (Fig. 1), with a median MIC of 4 mg/L and an interquartile range of 4–8 mg/L.

**Fig 2:**
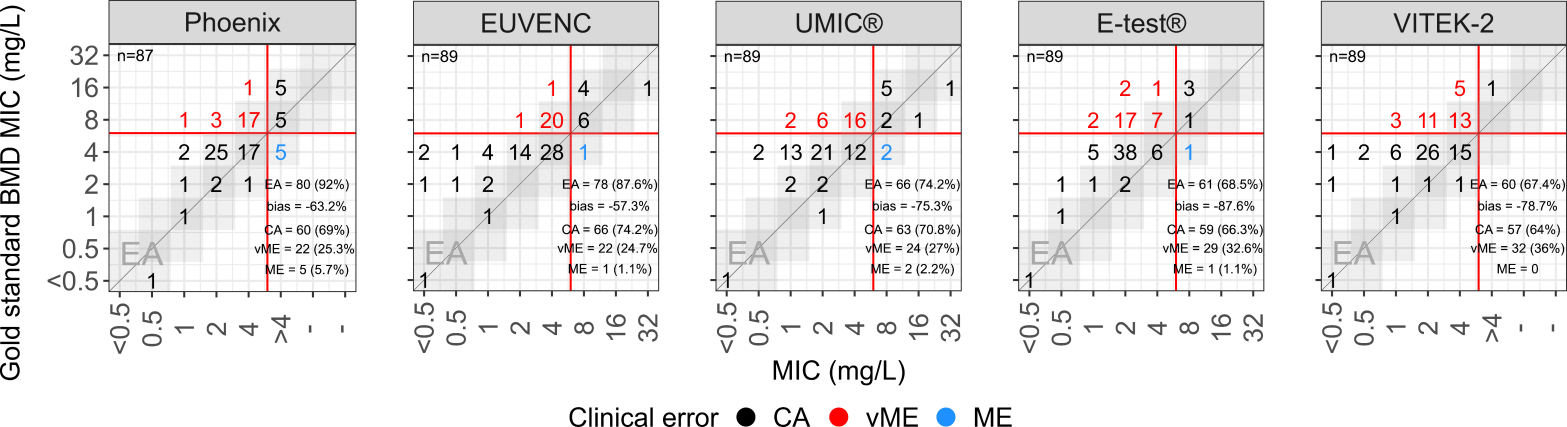
Commercial BMD and E-test versus gold standard BMD. MICs determined with the gold standard BMD method (reference) are on the *x*-axis. Phoenix/ EUVENC/UMIC/VITEK-2 and E-test MICs are on the *y*-axis. Isolates were categorized according to the gold standard BMD MICs and CLSI CBP. MICs/MICs are classified as CA (black), vME (red), and ME (blue). The red continuous lines denote the CLSI CBP. The gray areas in the top figures indicate essential agreement. EA, essential agreement; CA, categorical agreement; vME, very major error; ME, major error.

Among the tested methods, the Phoenix system was the most accurate, achieving an EA = 92% and a CA = 69%, with vMEs and MEs observed in 22 (25.3%) and 5 (5.7%) cases, respectively (Fig. 2). Two isolates repeatedly yielded invalid results and were excluded from the Phoenix performance calculations, leaving 87 evaluable isolates for that method. The EUVENC system showed an EA = 87.6% and a CA = 74.2%, with vME and MEs observed in 24.7% and 1.1% cases, respectively. The UMIC manual BMD method achieved an EA = 74.2% and a CA = 70.8%, with vME and MEs observed in 27% and 2.2% cases, respectively. The E-test exhibited a lower performance, with an EA = 68.5% and a CA = 66.3%, with vME and MEs observed in 32.6% and 1.1% cases, respectively. Finally, VITEK-2 performed the worst, with an EA = 67.4% and a CA = 64%, with vME and MEs observed in 36% and 0% cases, respectively. Notably, Phoenix, EUVENC, and UMIC correctly identified 10, 11, and 9 out of the 33 isolates with MICs above the CBP of 4 mg/L, respectively, whereas E-test and VITEK-2 detected only 4 and 1 such isolates, respectively.

## DISCUSSION

Reliable testing of daptomycin MIC is a challenge in routine diagnostics. None of the tested methods met the ISO-20776-2:2021 criteria (EA/CA ≥ 90%, bias ±30%) (12). All test methods exhibited substantial negative biases, suggesting a systematic underestimation of MICs. The commercial BMD methods, particularly the automated AST systems Phoenix and EUVENC, were the most accurate, achieving EA values ≥87.6%, which may still be considered satisfactory. The recently introduced UMIC manual BMD method also performed reasonably well, though its performance was behind the automated systems. All BMD methods consistently classified isolates with MIC values >8 mg/L as resistant. However, a significant proportion of isolates with MIC = 8 mg/L were misclassified due to underestimation, leading to erroneous susceptibility categorization. VITEK-2 and E-test were unsatisfactory, particularly in detecting isolates with MICs above the CBP. Worryingly, all methods displayed high negative biases, ranging from −57.3% for EUVENC to −87.6% for the E-test, linked to a general tendency to underestimate MICs. This underestimation is the primary cause of the high vME rates observed.

To our knowledge, this is the first study to systematically evaluate several commercially available AST methods for daptomycin susceptibility in VR*Efm*, including Phoenix and the recently marketed manual UMIC BMD strips. This study has some important limitations. First, the E-test was performed on only one commercial CAMH agar plate, although medium-dependent variability has been reported previously (10). Future studies should further include different CAMH agar plates. Second, the selection of VR*Efm* isolates with borderline susceptibility resulted in a relatively narrow MIC distribution, with a clear shift to higher MIC values (toward the epidemiological cut-off) compared to the distribution of a normal wild-type *E. faecium* population (Fig. 1). This, combined with the inherent variability in susceptibility testing and the use of a single MIC breakpoint (MIC > 4 mg/L classified as non-susceptible [no intermediate zone]), may have introduced bias in our study, leading to increased categorical disagreement between methods and potentially leading to several clinical errors. For these reasons, our findings cannot necessarily be extrapolated to predict overall assay performance for *E. faecium* with wild-type MIC distributions. On the other hand, this approach allowed us to focus specifically on isolates for which precise MIC determination is most critical.

MIC determination with commercially available methods remains challenging, particularly for values close to CBPs. Results obtained with VITEK-2 or E-test should be interpreted with caution. In laboratories using these systems, we recommend that MICs near the CBP (4–8 mg/L) be repeated and confirmed by BMD. Where confirmatory testing is not feasible, a laboratory comment should alert clinicians to the potential unreliability of results in this range and suggest discussion with an infectious diseases specialist if clinically indicated. This variability must be considered when making therapeutic decisions. Given these limitations and findings, a BMD-based MIC method is recommended for the reliable detection or confirmation of daptomycin resistance in VR*Efm*.

## SUPPLEMENTAL MATERIAL

Table S1 spectrum.03590-25-s0001.docx Strains used in the study and MICs determined by each method.

ASM does not own the copyrights to Supplemental Material that may be linked to, or accessed through, an article. The authors have granted ASM a non-exclusive, world-wide license to publish the Supplemental Material files. Please contact the corresponding author directly for reuse.
