# Supplementary material for: Evaluation of commercial susceptibility testing methods for in vitro determining daptomycin susceptibility in vancomycin-resistant Enterococcus faecium
Source: Microbiol Spectr. 2026 Mar 5;14(4):e03590-25. doi: 10.1128/spectrum.03590-25 (PMC13055354; doi:10.1128/spectrum.03590-25)
Supplement: Table S1 — Strains used in the study and MICs determined by each method. [file spectrum.03590-25-s0001.docx]

Supplementary data

Table S1. Strains used in the study and MICs determined by each method.

| **Study no.** | **Standard manual BMD** | **Phoenix** | **EUVENC** | **UMIC^®^** | **E-test^®^** | **VITEK-2** |
| --- | --- | --- | --- | --- | --- | --- |
|  | **MIC (mg/L)** | | | | | |
| 1 | 8 | 1 | 8 | 1 | 3 | 2 |
| 2 | 8 | 4 | 4 | 16 | 4 | 2 |
| 3 | 8 | 4 | 8 | 2 | 3 | 4 |
| 4 | 4 | 2 | 8 | 2 | 2 | 2 |
| 5 | 4 | 2 | 4 | 2 | 2 | 2 |
| 6 | 4 | 2 | 4 | 4 | 3 | 2 |
| 7 | 4 | 2 | 2 | 1 | 2 | 2 |
| 8 | 2 | 2 | 0.5 | 1 | 1.5 | 2 |
| 9 | 4 | 4 | 0.25 | 2 | 2 | 0.5 |
| 10 | 4 | 4 | 1 | 2 | 3 | 1 |
| 11 | 2 | 1 | 0.25 | 2 | 0.5 | 1 |
| 12 | 0.25 | 0.5 | 0.25 | 0.25 | 0.25 | 0.125 |
| 13 | 2 | 2 | 1 | 2 | 1 | 0.25 |
| 14 | 4 | 2 | 1 | 2 | 1.5 | 2 |
| 15 | 4 | 2 | 0.25 | 0.5 | 1 | 0.5 |
| 16 | 4 | 1 | 1 | 2 | 1 | 2 |
| 17 | 4 | 2 | 4 | 4 | 1.5 | 2 |
| 18 | 8 | 2 | 4 | 4 | 1.5 | 2 |
| 19 | 4 | 2 | 0.5 | 4 | 1.5 | 4 |
| 20 | 4 | 2 | 4 | 4 | 1.5 | 2 |
| 21 | 1 | 1 | 1 | 2 | 0.5 | 1 |
| 22 | 8 | 8 | 4 | 8 | 2 | 4 |
| 23 | 4 | 8 | 4 | 8 | 3 | 4 |
| 24 | 4 | 2 | 4 | 2 | 2 | 4 |
| 25 | 4 | 2 | 4 | 4 | 2 | 2 |
| 26 | 4 | 2 | 4 | 2 | 2 | 4 |
| 27 | 4 | 2 | 4 | 4 | 2 | 4 |
| 28 | 2 | 4 | 1 | 1 | 2 | 4 |
| 29 | 8 | 4 | 4 | 2 | 2 | 1 |
| 30 | 4 | 4 | 4 | 4 | 2 | 4 |
| 31 | 4 | 8 | 4 | 8 | 3 | 4 |
| 32 | 4 | 4 | 2 | 4 | 2 | 4 |
| 33 | 8 | 4 | 4 | 4 | 1.5 | 2 |
| 34 | 8 | 4 | 4 | 4 | 2 | 4 |
| 35 | 8 | 4 | 4 | 4 | 2 | 2 |
| 36 | 8 | 4 | 4 | 4 | 2 | 4 |
| 37 | 8 | 8 | 4 | 4 | 3 | 4 |
| 38 | 4 | 8 | 4 | 4 | 3 | 4 |
| 39 | 4 | 2 | 4 | 1 | 2 | 4 |
| 40 | 8 | 4 | 4 | 2 | 2 | 2 |
| 41 | 4 | 2 | 2 | 1 | 1.5 | 2 |
| 42 | 4 | 2 | 2 | 1 | 1.5 | 2 |
| 43 | 4 | 4 | 4 | 2 | 2 | 2 |
| 44 | 4 | 2 | 4 | 1 | 2 | 4 |
| 45 | 8 | 4 | 8 | 4 | 2 | 2 |
| 46 | 4 |  | 4 | 1 | 1.5 | 4 |
| 47 | 4 | 4 | 2 | 2 | 2 | 2 |
| 48 | 4 | 4 | 4 | 2 | 2 | 2 |
| 49 | 8 | 8 | 4 | 4 | 3 | 4 |
| 50 | 4 | 2 | 2 | 2 | 1.5 | 1 |
| 51 | 4 | 4 | 4 | 2 | 2 | 2 |
| 52 | 4 | 2 | 2 | 1 | 1.5 | 1 |
| 53 | 4 | 2 | 4 | 1 | 1.5 | 1 |
| 54 | 4 | 4 | 2 | 2 | 2 | 2 |
| 55 | 4 | 4 | 4 | 4 | 3 | 4 |
| 56 | 8 | 4 | 4 | 2 | 2 | 4 |
| 57 | 4 | 4 | 4 | 2 | 2 | 4 |
| 58 | 4 | 4 | 2 | 2 | 2 | 2 |
| 59 | 4 | 2 | 2 | 0.5 | 0.75 | 1 |
| 60 | 4 | 2 | 1 | 1 | 1.5 | 2 |
| 61 | 4 | 4 | 4 | 2 | 2 | 2 |
| 62 | 4 | 4 | 4 | 2 | 2 | 2 |
| 63 | 4 | 4 | 4 | 2 | 2 | 2 |
| 64 | 4 | 4 | 2 | 2 | 2 | 2 |
| 65 | 8 | 4 | 4 | 2 | 1.5 | 2 |
| 66 | 4 | 2 | 4 | 1 | 1.5 | 2 |
| 67 | 8 | 4 | 4 | 4 | 6 | 1 |
| 68 | 4 | 8 | 4 | 4 | 6 | 4 |
| 69 | 16 | 8 | 8 | 8 | 8 | 4 |
| 70 | 16 | 8 | 8 | 8 | 6 | 4 |
| 71 | 16 | 8 | 32 | 32 | 8 | 8 |
| 72 | 16 | 4 | 8 | 8 | 2 | 4 |
| 73 | 16 | 8 | 4 | 8 | 2 | 4 |
| 74 | 8 | 4 | 2 | 4 | 2 | 4 |
| 75 | 8 | 4 | 8 | 4 | 2 | 2 |
| 76 | 4 | 8 | 2 | 4 | 2 | 2 |
| 77 | 4 | 2 | 4 | 1 | 2 | 2 |
| 78 | 8 | 4 | 8 | 4 | 2 | 4 |
| 79 | 8 |  | 4 | 2 | 2 | 4 |
| 80 | 8 | 4 | 8 | 4 | 3 | 4 |
| 81 | 4 | 1 | 2 | 2 | 1.5 | 0.25 |
| 82 | 8 | 8 | 4 | 4 | 2 | 2 |
| 83 | 8 | 2 | 4 | 1 | 1.5 | 1 |
| 84 | 4 | 2 | 2 | 1 | 1 | 1 |
| 85 | 8 | 8 | 4 | 8 | 3 | 4 |
| 86 | 8 | 2 | 4 | 4 | 0.75 | 2 |
| 87 | 16 | 8 | 8 | 8 | 3 | 4 |
| 88 | 8 | 4 | 4 | 4 | 1 | 4 |
| 89 | 4 | 4 | 4 | 1 | 1 | 2 |
| *E. faecalis* ATCC 29212 | 4 | 1 | 2 | 4 | 1.5 | 4 |
| *E. faecalis* ATCC 29212 | 4 | 1 | 4 | 4 | 1.5 | 4 |
| *E. faecalis* ATCC 29212 | 4 | 1 | 2 | 4 | 2 | 4 |
